# Solid-liquid density and spin crossovers in (Mg, Fe)O system at deep mantle conditions

**DOI:** 10.1038/srep37269

**Published:** 2016-11-22

**Authors:** Dipta B. Ghosh, Bijaya B. Karki

**Affiliations:** 1 School of Electrical Engineering and Computer Science, Louisiana State University, Baton Rouge, LA 70803; 2 Department of Geology and Geophysics, Louisiana State University, Baton Rouge, LA 70803; 3 Center for Computation and Technology, Louisiana State University, Baton Rouge, LA 70803.

## Abstract

The low/ultralow-velocity zones in the Earth’s mantle can be explained by the presence of partial melting, critically depending on density contrast between the melt and surrounding solid mantle. Here, first-principles molecular dynamics simulations of (Mg, Fe) O ferropericlase in the solid and liquid states show that their densities increasingly approach each other as pressure increases. The isochemical density difference between them diminishes from 0.78 (±0.7) g/cm^3^ at zero pressure (3000 K) to 0.16 (±0.04) g/cm^3^ at 135 GPa (4000 K) for pure and alloyed compositions containing up to 25% iron. The simulations also predict a high-spin to low-spin transition of iron in the liquid ferropericlase gradually occurring over a pressure interval centered at 55 GPa (4000 K) accompanied by a density increase of 0.14 (±0.02) g/cm^3^. Temperature tends to widen the transition to higher pressure. The estimated iron partition coefficient between the solid and liquid ferropericlase varies from 0.3 to 0.6 over the pressure range of 23 to 135 GPa. Based on these results, an excess of as low as 5% iron dissolved in the liquid could cause the solid-liquid density crossover at conditions of the lowermost mantle.

Density perhaps is the single most important property controlling the stability and mobility of melts such as the ones expected above and below the mantle transition zone and at the bottom of the lower mantle^1^,^2^. Whether melt will ascend or descend in a partially molten mantle at a depth depends on density contrast between the melt and the surrounding solid mantle^3^,^4^,^5^,^6^,^7^. Negatively buoyant (sinking) melts imply that a high degree of partial melting or a basal magma ocean may be present in the deepest parts of the mantle^8^,^9^. Such melts trapped above the core-mantle boundary may be enriched in Fe and may have a higher MgO/SiO_2_ ratio than the solid mantle^10^,^12^. Improved knowledge about the densities of silicates and oxides in their solid and molten states is thus critical to our understanding of the mantle stratification and dynamical behavior.

Relevant solid-melt density contrasts over most of the lower mantle pressure regime still remain unknown. A material in crystalline state is denser than its liquid state because of the difference in the atomic packing between the two states. The difference diminishes considerably with compression, however, the density crossover may not occur even at very high pressure. For instance, the magnitude of the crystal-liquid density gap for MgO periclase −a major lower mantle material decreases initially rapidly with pressure, and then gradually at high pressure^13^. This trend holds true for other mantle materials because their crystalline phases undergo pressure-induced structural transition to one or more successive denser phases with increasing pressure^14^,^15^. Moreover, these materials expand on heating, more so in the liquid state. How temperature influences their densities also depends on pressure because the thermal pressure in the liquid changes with compression differently than in the solid^14^,^16^. The molten state perhaps always remains less dense than the solid state thereby ruling out the possibility of crystal-liquid density crossover in any iso-chemical situation of mantle relevance solely because of compression and thermal expansion.

Another key factor that can influence the density contrasts is compositional difference between the solid and liquid states. Iron perhaps is the most important element because it is heavier than other common mantle elements and also undergoes pressure-induced electronic spin transition, both affecting the density^17^,^18^. If the liquid could accommodate more iron than the solid, a solid-to-liquid density inversion would be possible^11^,^12^,^19^. However, we lack direct quantitative assessment of the effects of iron on the solid and liquid state densities of any major mantle material at high pressure. Complementary to difficult experimentation is the first principles computation, which was previously used in the study of mantle materials, including Fe-bearing solids^19^,^20^,^21^,^22^. Here, we report the results from the density functional theory-based simulations of (MgFe)O ferropericlase solid and liquid systems with up to 25% iron concentration using the generalized-gradient approximation (GGA) and projector-augmented wave method as implemented in the VASP program^23^ (Methods, Supplementary Fig. 1). Our first-principles molecular dynamics (FPMD) simulations sampled the temperature range of 3000 to 8000 K and the pressure range of 0 to 140 GPa. The solidus temperature of the lower mantle at pressures near the core-mantle boundary is expected to be in the range of 3570–4200 K based on chondritic^24^ or pyrolitic^25^ mantle models. The corresponding liquidus temperature lies above the solidus temperature by 700 K or more^24^,^26^. The calculated thermal equation of state and iron spin states help us answer some key questions: How is Fe-induced change in the density sensitive to pressure and temperature? Does Fe undergo spin transition in liquid ferropericlase? Does Fe prefer to partition in the liquid over the solid? How do the crystal and liquid densities compare over the entire lower mantle pressure regime for different compositions? As such, we aim to determine how much excess iron of the melt may actually cause the crystal-liquid density crossover to occur in (Mg,Fe)O ferropericlase system at deep mantle conditions.

## Results

**Figure 1:**
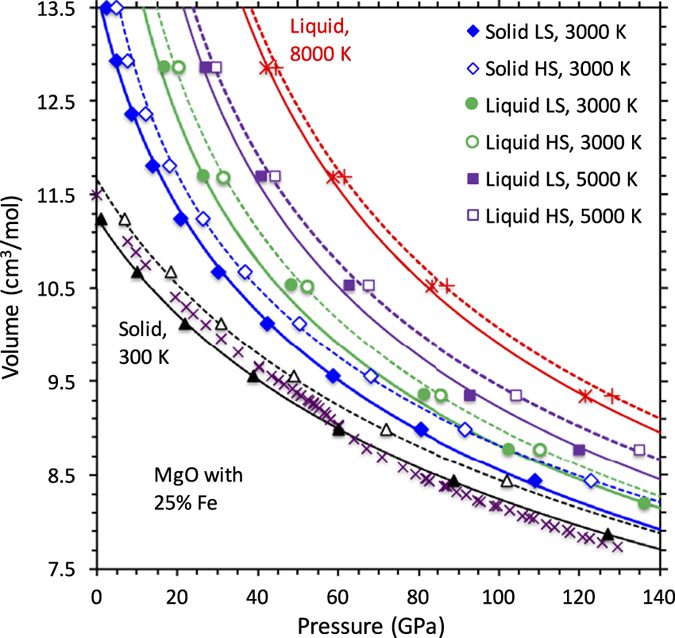
Pressure-volume relationships for (Mg_0.75_, Fe_0.25_)O liquid and solid. The calculated results are shown for high spin, HS (open symbols) and low spin, LS (solid symbols) iron along different isotherms: both liquid and solid states at 3000 K, and only liquid state at 5000 and 8000 K. Also shown are the 300 K solid results (triangles) compared with the experimental data^17^,^29^. The curves represent the equation of state fits using Equation 2.

We consider two spin phases of (Mg, Fe)O liquid: The one with all Fe ions in low-spin (LS) is a non-magnetic phase and the other with all Fe ions in high-spin (HS) is a magnetic phase. The calculated pressure-volume curves of the LS ferropericlase liquid systematically lie below the corresponding HS curves (Fig. 1) so the low-spin phase shows higher density relative to the high-spin phase. For instance, the volume difference between two spin phases for 25% Fe concentration along the 3000 K isotherm decreases from about 0.6 cm^3^mol^−1^ at zero pressure to 0.3 cm^3^mol^−1^ at 100 GPa. They are somewhat smaller than those for solid ferropericlase (Fig. 1). In both cases, the spin associated volume differences are nearly insensitive to temperature and iron concentration.

**Figure 2:**
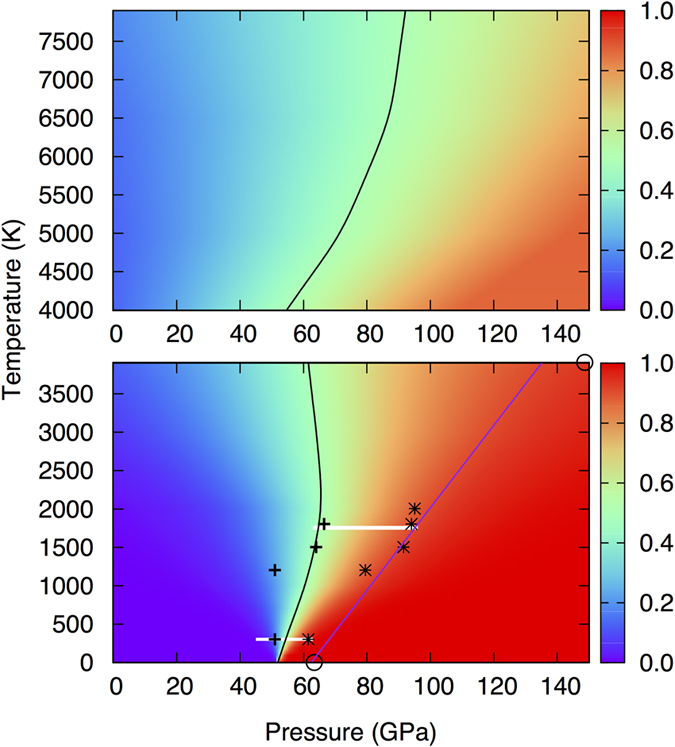
Spin phase diagrams. The calculated high spin-low spin crossovers of iron in the (Mg_0.75_Fe_0.25_)O liquid (top) and solid (bottom). The vertical color scale represents the fraction (*n*) of the low-spin state (LS) calculated using Equation 1. The value of *n* varies from 0 (i.e., 100% high-spin state) to 1 (i.e., 100% low-spin state). The black curves represent the *n* = 0.5 isolines, that is, the mid points of the spin crossovers in the *P-T* space. Also shown for solid ferropericlase are the experimental data (pluses^27^, asterisks^29^, horizontal white lines^28^) and the previous GGA + *U* calculations (blue line)^30^ along with the present GGA + *U* results (circles).

Our FPMD simulations show that iron undergoes a pressure-induced high-spin to low-spin (electronic spin-pairing) transition in both solid and liquid ferropericlase (Fig. 2), as also found by many previous experiments^17^,^27^,^28^,^29^ and calculations^20^,^22^,^30^,^31^. The spin phase diagram can be calculated in terms of the fraction of low spin state (*n*) given^20^,^29^,^31^ by


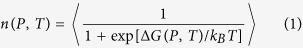


Here, Δ*G* = [(*H*_LS_ − *H*_HS_) − *T*Δ*S*] represents the molar Gibbs free energy difference between the low-spin and high-spin ferropericlase and 〈〉 denotes time average. The enthalpy values come directly from the FPMD simulations. The electronic and magnetic entropies are also obtained from the simulations (Supplementary text). The predicted spin transition is sharp at 0 K occurring at 52 GPa so that all Fe ions are in the high-spin state (total spin *S* = 2) on low-pressure side and all in the low-spin state (*S* = 0) on high-pressure side. The transition pressure is bounded by previous calculations from the above^22^ and below^20^,^31^. However, the transition at elevated temperatures becomes gradual exhibiting a pressure interval of coexisting LS and HS states with their respective proportions, *n* and (1 − *n*) determined by Equation 1. Temperature systematically widens the stability field of the mixed-spin phase toward higher pressures (Fig. 2, bottom). Our calculated spin phase diagram for solid (Mg, Fe)O agrees with the available experimental data within their scattered range^17^,^27^,^28^,^29^.

Our simulations also show that a gradual high-spin to low-spin transition occurs in liquid ferropericlase over a finite pressure interval about 55 GPa at 4000 K (Fig. 2, top). The spin crossover pressure is positively correlated with temperature: The transition pressure defined by *n* = 0.5 (that is, the middle point of the crossover) increases and the transition zone broadens with increasing temperature. Remarkably, the spin phase diagram of the liquid ferropericlase (Fig. 2, top) almost matches with that of the solid ferropericlase (Fig. 2, bottom) along the 4000 K isotherm. The populations of LS states are comparable between the solid and liquid phases, being about 50% around 60 GPa. This correspondence actually exists over an extended pressure range, with low-spin populations being around 80 and 90% at 100 and 135 GPa, respectively. Our results thus suggest that liquid (Mg,Fe)O like its solid counterpart should be in a mixed-spin phase with iron ions perhaps mostly in the low-spin state at pressures corresponding to the deep parts of the lower mantle.

It is noted that the spin phase diagrams obtained here for both solid and liquid ferropericlase show notable differences with those from the recent FPMD calculations^22^,^30^. Interestingly, the discrepancies could be largely attributed to the use of the Hubbard *U* term (Supplementary Fig. 2). Our GGA + *U* test simulations show that the inclusion of *U* shifts the HS-LS transition pressure in solid ferropericlase from 52 to 63 GPa at 0 K. The pressure shift becomes larger at higher temperature. The increased transition pressures corresponding to *n* = 0.5 at 0 and 4000 K are comparable with the previous GGA + *U* pressures^22^ (Fig. 2, bottom). For ferropericlase liquid, the effects of *U* are so large that the pressure of co-existing HS and LS in equal proportions goes beyond 150 GPa at 6500 and 8000 K, again being consistent with the previous GGA + *U* prediction^30^. The difference in the Clapeyron slopes for the spin crossovers between the present and previous studies (Fig. 2) can be attributed to mainly the entropy factors (Supplementary Fig. 3). Unlike the spin phase diagram, the *U*-term does not affect the density significantly as discussed later (Supplementary Fig. 4).

**Figure 3:**
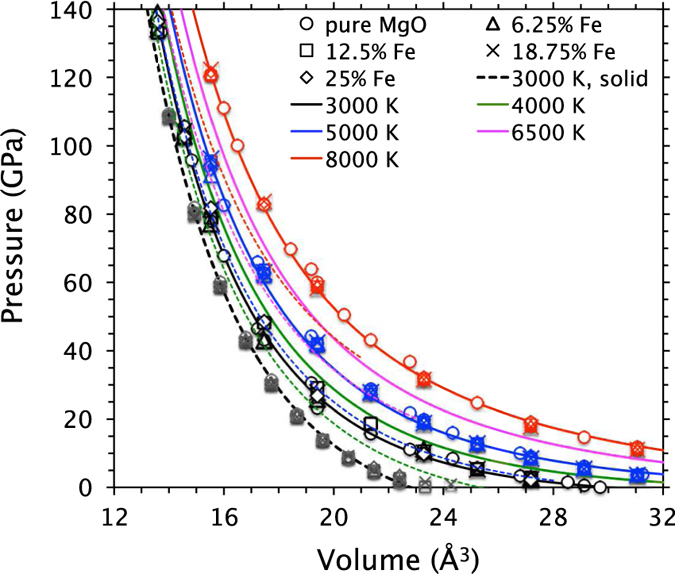
Equation of state isotherms of the (Mg, Fe)O liquid system with low spin iron. Symbols represent the pure phase and different Fe concentrations (6.25, 12.5, 18.75 and 25%). Solid lines represent the composition-averaged fits for each temperature obtained using Equation 2 in which the two coefficients are: 
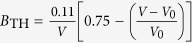
 and 
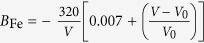
, where *V*_0_ = 30 Å^3^. Also shown are the solid results for different Fe concentrations at 3000 K (grey symbols and dashed line), and the fits at higher temperatures (dashed lines).

The calculated pressure-volume-temperature (*P* − *V* − *T*) results (Fig. 3) for all low spin iron-bearing liquids can be described with one single equation of state:


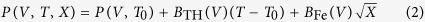


The first term (Equation 2) represents the reference isotherm (*T*_0_ = 3000 K) of pure MgO liquid taken to be the third order Birch-Murnaghan equation: *V*_0_ (Å^3^) = 30.0, *K*_0_(GPa) = 16.6, 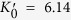. For all concentrations of low spin iron, the pressure-volume results almost match with those of the pure liquid. The calculated thermal contributions are the same within the computational uncertainty for pure and alloyed melts. They all increase considerably with compression thus requiring a strong volume-dependent coefficient *B*_TH_. This behavior is in sharp contrast to the case of the solid phase where the thermal pressure is weakly dependent on volume^13^,^14^,^32^. The thermal contributions in the liquid are systematically larger than those in the solid at most compressed volumes. The effects of low-spin iron on pressure are small, compared to the high-spin iron effects (Supplementary Fig. 5). The corresponding coefficient (*B*_Fe_) in Equation 2 changes from slight negative to positive at high compression.

**Figure 4:**
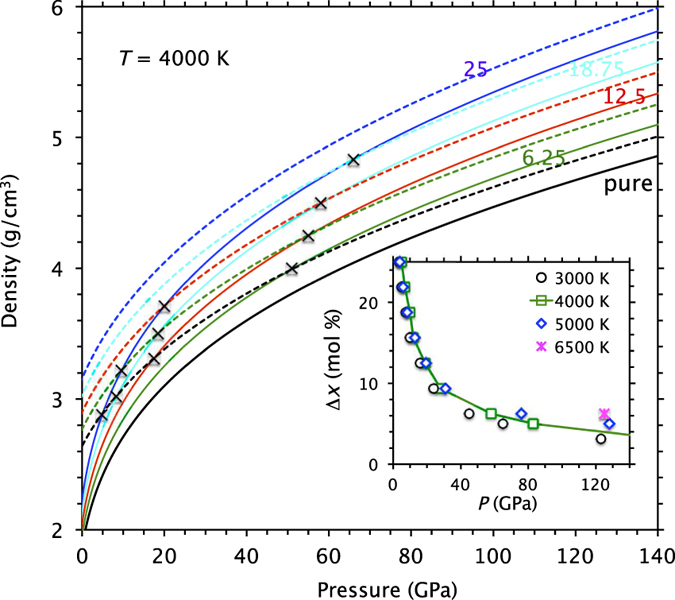
Density-pressure profiles of (Mg,Fe)O liquid and solid at 4000 K. The liquid (solid lines) and solid (dashed lines) densities are shown as a function of pressure for iron concentrations of 6.25, 12.5, 18.75 and 25.0% (shown by the corresponding numbers). The lines from the bottom (black solid and dashed lines) to the top denote the pure systems to increasing concentration with possible density crossovers marked. Comparisons are also made for additional concentrations (Supplementary Fig. 6). The inset shows the estimated excess iron content (∆*X*) of the liquid state (from Equation 3) required for the solid-liquid density crossover to occur in (Mg, Fe)O system as a function of pressure at different temperatures.

The calculated density-pressure isotherms for the pure and Fe-bearing liquids remain mostly parallel with each other (Fig. 4). So do the calculated solid density-pressure profiles. However, the isotherms are not parallel between the liquid and solid phases due to large difference in their compressibility. The liquid density increases more rapidly compared to the solid density with compression, and two densities approach each other as pressure increases. At the ambient pressure and 3000 K, the crystal-liquid density difference (∆ρ) is large (0.71 g/cm^3^ for the pure phases). As pressure increases, the density gap decreases rapidly initially (0.25 g/cm^3^ at 23 GPa and 3000 K) and then more gradually at high pressure (0.14 g/cm^3^ at 135 GPa and 4000 K). Temperature widens the density gap somewhat because the liquid has larger thermal pressure (and hence larger thermal expansion) than the solid as our simulations have found. The gap widening at higher iron content has origin in the mass itself. For 25% Fe, the calculated values of ∆ρ are 0.84, 0.30, and 0.18 g/cm^3^, respectively, at 0, 23, and 135 GPa. Our first-principles findings thus confirm that any iso-chemical solid-liquid density gap remains positive finite under conditions of the entire mantle.

## Discussion

The density-pressure profiles of the liquid and solid phases for different (Mg, Fe)O compositions intersect with each other implying the possibility of solid-liquid density crossover (Fig. 4, Supplementary Fig. 6). Using these results, we can estimate the pressure at which the density of the liquid phase with a certain excess amount (∆*X*) of iron dissolved in the liquid exceeds the solid density. This density inversion condition is defined by


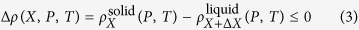


where *X* is the iron content of the solid (Mg_1*−X*_Fe_*X*_)O phase. Corresponding to four iron concentrations that we simulated in this study, we can consider ∆*X* values of 6.25, 12.5, 18.75 and 25%. Each value has more than one instance thereby making multiple pressure estimates possible, for instance, ∆*X* of 6.25% has four comparable estimates. Thus derived ∆*X-*pressure profiles along different isotherms (Inset of Fig. 4) show that the critical excess iron content (∆*X*) of the liquid decreases rapidly initially with increasing pressure, dropping below 10% after 20 GPa and then below 5% after 100 GPa. A higher ∆*X* value of 20% can cause the liquid-solid density crossover in ferropericlase at pressures as low as 10 GPa, as also found by the recent GGA + *U* calculations along the liquidus^30^.

We now discuss the relevance of the estimated excess iron content (∆*X*) for the density crossover to occur in ferropericlase by considering Fe partitioning between the solid and liquid states. The partition coefficient can be calculated from energetics of the following reaction:


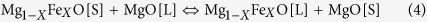


where Fe-bearing solid [S] and pure liquid [L] react to form Fe-bearing liquid [L] and pure solid [S]. The Gibbs free energy per mol of FeO can be written as


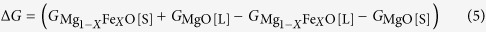


The equation 5 requires full knowledge of the Gibbs free energy, *G* = *H* *−* *TS* of all systems. The enthalpy *H* can be readily calculated from our simulations. The calculation of the entropy *S* is intensive, requiring the thermodynamics integration method^30^,^33^, which was not used in this study. However, it is likely that the vibrational and configuration contributions from different phases in the above reaction (Equation 4) will largely cancel out. Considering the same (low) spin state of all Fe atoms for the solid and liquid ferropericlase, we can also ignore the magnetic entropy. The net electronic contribution to the entropy calculated from the simulations turns out to be small and is included. Based on these arguments, it makes sense to use the enthalpy term only^34^, which was calculated as a function of pressure for different iron concentrations. The estimated value of ∆*G* is 0.40 to 0.18 eV over the pressure range (23 to 135 GPa) of the lower mantle.

The following thermodynamic relation defines the iron partitioning between the solid and and liquid ferropericlase:


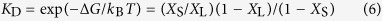


For ∆*G* of 0.40 and 0.18 eV, *K*_D_ values at 4000 K are 0.30 to 0.57, respectively. Thus estimated partition coefficient results are lower than the only available experimental data of 0.63 at 22–24.5 GPa for ferropericlase^35^. The large difference between the computed and measured results may be largely due to the neglect of the entropy contributions in this study and also due to experimental uncertainties. For comparisons, the measured data for (Mg, Fe)SiO_3_ system are 0.3–0.4 at 25 GPa, dropping below 0.1 above 75 GPa^11^,^36^. Measurements on Al-bearing silicates have been controversial, reporting large values^37^ such as 0.47 at 110 GPa. Nevertheless, our estimated *K*_*D*_ for ferropericlase lying below unity implies that iron goes preferentially into the liquid. Considering a bulk 10% FeO content of a partially melted system, the liquid state should contain 17 and 6% excess FeO, respectively, for the *K*_D_ values of 0.30 and 0.57. These excess iron contents are somewhat larger those based on the Equation 3. Thus, our estimated excess iron contents for the solid-liquid density inversion at deep mantle pressures are feasible with preferred partitioning of iron to the melt.

Based on our calculated spin phase diagrams (Fig. 2), the solid and liquid phases likely contain low spin iron in similar amounts at any high pressure. Even if the spin transition zones may not match between the solid and liquid phases as found by the GGA + *U* calculations^30^, the transitions are very broad so each phase would still be in a mixed-spin state. This means that the spin transitions cannot impact the possible crystal-liquid density crossover significantly. By considering the pressure, temperature, and spin factors together for the (Mg, Fe)O system for several concentrations, our analysis shows that the excess iron content of ferropericlase melt for it to be denser than the solid phase can be as low as 5% at conditions of the lowermost mantle. Small amounts of excess iron (∆*X*) mean that the dense melts may also see preferred partitioning of lighter incompatible elements like H, He, S, etc. The deepest part of the Earth’s mantle perhaps existing as either a deep-seated liquid generated in contact with hot core or a residual dense magma ocean can be enriched in minor and trace elements as previously suggested^38^,^39^.

## Methods

First principles molecular dynamics simulations of (Mg, Fe)O ferropericlase were performed using spin-polarized GGA^40^ and projector augmented wave method^41^. Simulations based on the canonical (*NVT*) ensemble were performed over a wide volume (*V*) range resulting in pressure range of 0 to 140 GPa at temperature (*T*) from 3000 to 8000 K. The number (*N*) of atoms used were 64 for the small supercell with which most simulations were performed, compared to the large supercell (216 atoms) that was used to assess the finite system-size effects. Four iron concentrations including 6.25, 12.5, 18.75 and 25% were considered. For each composition, the initial atomic configuration was first melted at high temperature (6000 to 10000 K depending on the volume) and then quenched/cooled down to desired lower temperatures subsequently. The run durations varied from 10 to 100 ps (with a time step of 1 femtosecond used), depending on the volume and temperature condition of the simulation. Averages of the energy and pressure were computed by the blocking method^42^. The liquid state of the simulated system was assured by examining the mean square displacement and radial distribution function plots. The Pulay stress arising from the use of a finite cutoff of 400 eV at *Γ* point was added. Further details can be found elsewhere^5^,^13^.

For systems with correlated (*d* or *f*) electrons, a correction formally known as Hubbard *U* to the self-consistent solution is usually included in order to better describe the electronic structure features, e.g., opening up of band gap, better experimental agreement of the band width, particularly at the ambient conditions. The appropriateness of the *U* term at elevated conditions of pressure and temperature is not well founded, however. We have also performed a few GGA + *U* calculations for the comparison purpose. They showed that the effects of the *U* term^43^ on the equation of state and density are small (Supplementary Fig. 1 and Fig. 4). The effects on the electronic density of states and the spin-crossovers are substantial (Supplementary Figs 7–9), being mostly consistent with the recent GGA + *U* calculations^22^,^30^. As far as the density differences between the solid and liquid phases are concerned, they appear to largely be insensitive to the choice of GGA/GGA + U at all conditions (Supplementary Fig. 4). So are the density differences between the high spin and low spin Fe-bearing ferropericlase.

## Additional Information

**How to cite this article**: Ghosh, D. B. and Karki, B. B. Solid-liquid density and spin crossovers in (Mg, Fe)O system at deep mantle conditions. *Sci. Rep.* **6**, 37269; doi: 10.1038/srep37269 (2016).

**Publisher’s note:** Springer Nature remains neutral with regard to jurisdictional claims in published maps and institutional affiliations.

## Supplementary Material

Supplementary Information
