# Supplementary material for: Solid-liquid density and spin crossovers in (Mg, Fe)O system at deep mantle conditions
Source: Sci Rep. 2016 Nov 22;6:37269. doi: 10.1038/srep37269 (PMC5118715; doi:10.1038/srep37269)
Supplement: Supplementary Information [file srep37269-s1.pdf]

Supplementary Information

Solid-liquid density crossover in (Mg,Fe)O system at deep mantle conditions
Dipta B Ghosh and Bijaya B. Karki

Supplementary figure 1: Calculated pressure-volume relationships

The GGA results (open and solid triangles for high- and low-spin Fe bearing systems) at 300 K are compared with the experimental data[1] (pluses). Also shown are the static results from GGA+U and LDA simulations for the both spin states. As usually expected, GGA overestimates the volume whereas LDA underestimates it thereby bounding the experimental data[1] from the above and below, respectively. The effect of *U* is to slightly increase the volume so the both HS and LS curves are shifted upwards somewhat relative to GGA results. All these systematic effects essentially cancel out when the density differences between the solid and liquid states are considered. For instance, the volume collapse at the spin transition remains nearly the same with GGA, GGA+U, and LDA methods.

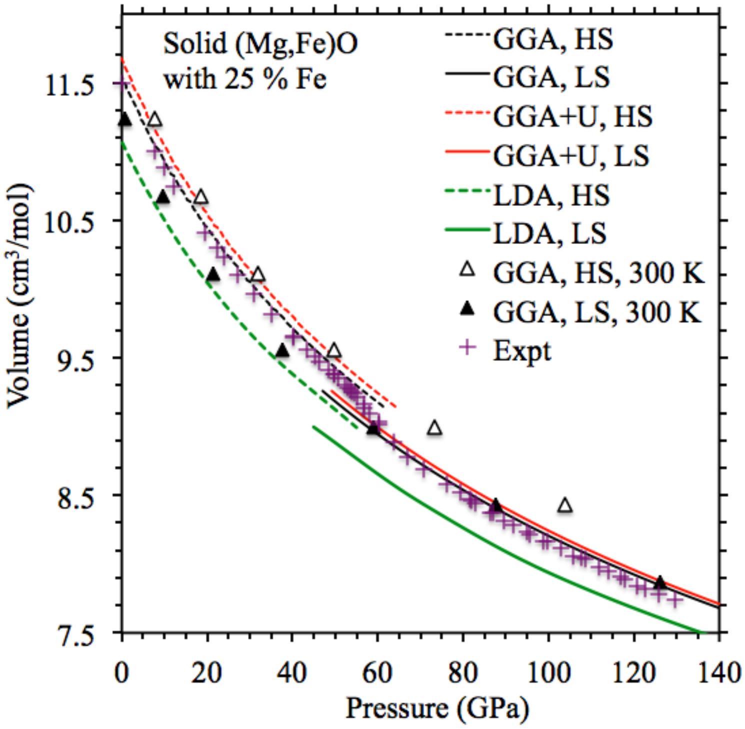

Supplementary figure 2. **Effects of the magnetic entropy choice and the U term on the spin phase diagram for the solid ($(Mg_{0.75}Fe_{0.25})O$)**

The high-spin to low-spin phase boundary represented here by the $n = 0.5$ isoline (connecting the mid-points in the crossover region at which the fractions of LS and HS states are equal) shown in Fig. 2 (bottom) is the S2 (blue) curve obtained using the magnetic moments of all atoms (Eq. 1). (See Supplementary text for the entropy equations). The S1 (green) and S3 (red) curves correspond to the use of the ideal magnetic entropy without (Eq. 3) and with (Eq. 4) orbital degeneracy of the t_{2g} states, respectively. The symbols represent our GGA+U (with U = 2.5 eV) test calculations at 0 and 4000 K (circles and squares), which compare favorably with the $n = 0.5$ boundary extracted from the previous GGA+U calculations[2] (straight purple line) using Eq. 3. The experimental data are shown by the horizontal brown line/bar[3], asterisk[1] and plus[4]. Remarkably, the GGA and GGA+U calculations appear to bound the experimental data from the below and above, respectively. The effect of U is to shift the transition to higher pressure, much more at higher temperature. Inappropriate U values can lead to situations of vanishing of the spin transition[5].

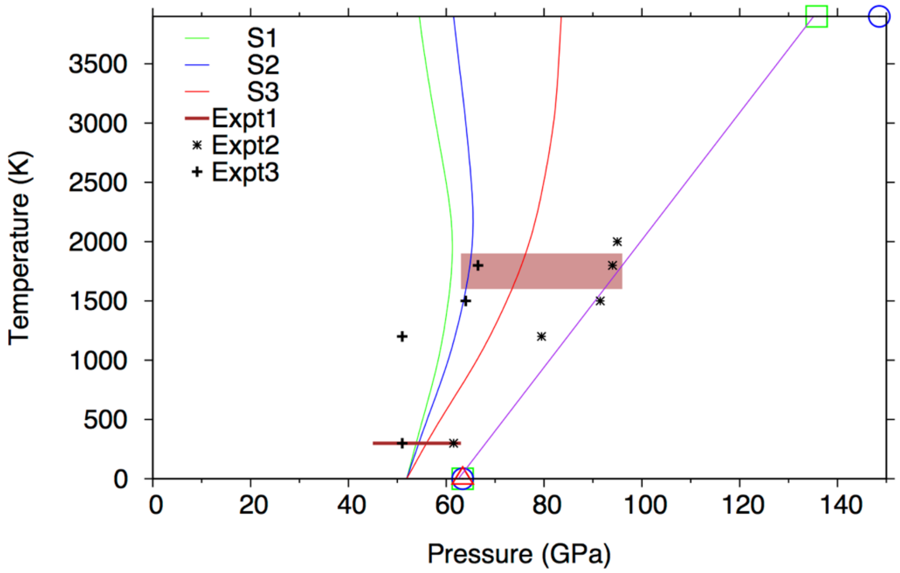

Supplementary figure 3. Entropy contributions and the spin phase diagram for the liquid $(Mg_{0.75}Fe_{0.25})O$

The pressures of n = 0.5 (the mid-point in the crossover region) at 6500 and 8000 K are shown for GGA (solid lines with solid symbols) and GGA+U (dashed lines with open symbols) by considering the enthalpy only (triangles) and both the enthalpy and entropy (squares). The enthalpy-based transition pressure decreases with temperature for both types of calculations (solid and open triangles). The electronic and magnetic entropy values compete with each other favoring LS and HS states, respectively. The net entropy contribution (circles) is positive, slightly decreasing with temperature for GGA whereas it changes from negative at 6500 K to positive at 8000 K for GGA+U. (Note that for comparison purpose, the calculated magnetic moments for iron ions only were used here as in the previous study[6].) So, the GGA-based transition pressure (solid squares) is positively correlated with temperature, as also shown in Fig 2, top. The GGA+U calculation shifts the transition higher pressures (open squares) relative to GGA calculation (filled symbols) at both temperatures, and the shifted pressure actually decreases with increasing temperature. Thus, the predicted Clapeyron slope for the spin phase boundary for liquid ferropericlase is positive for GGA but it can be negative for GGA+U as also found by the previous study[6].

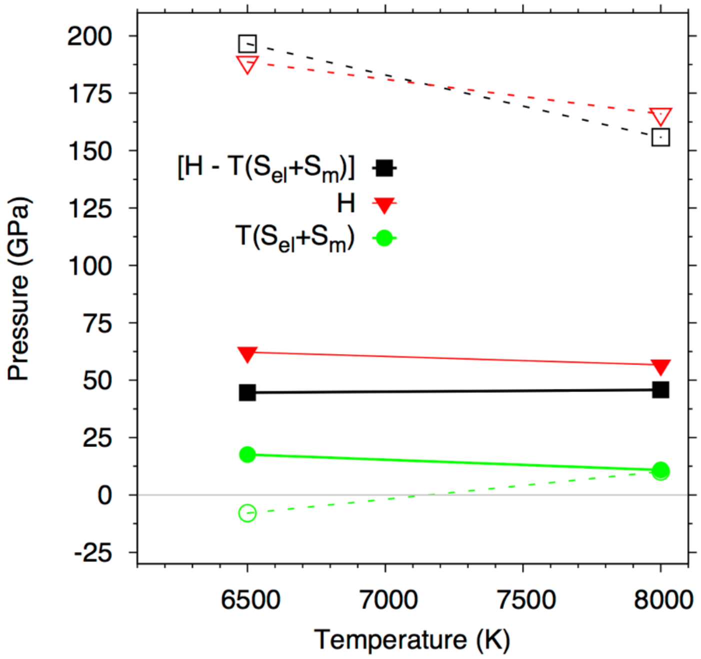

Supplementary figure 4: Density comparisons with and without Hubbard U

The calculated density-pressure profiles of (Mg,Fe)O solid at 0 K for GGA and GGA+U for two U values (*top*). The density curves almost overlap with each other. The density differences associated with U for solid (at 0 and 4000 K) and liquid (at 6500 K) ferropericlase in both high-spin (HS) and low-spin (LS) states (*bottom*). The differences are small, lying within 0.05 g/cm^3. This means that the density contrasts between the solid and liquid remain nearly insensitive to the choice of GGA/GGA+U at all conditions. So are the HS-LS density differences.

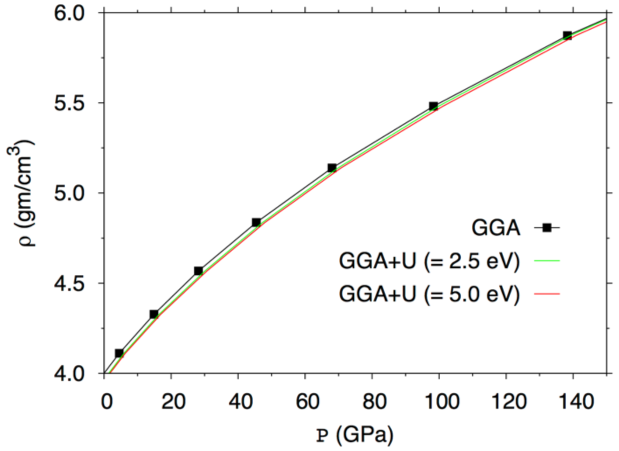

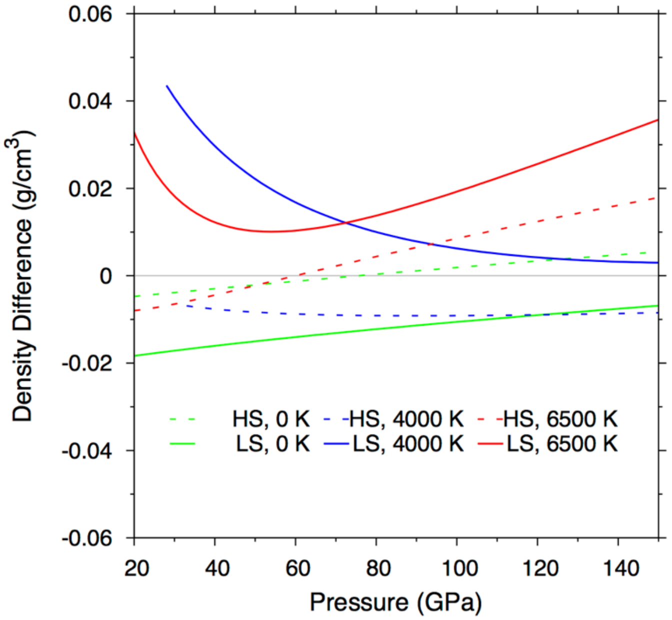

Supplementary figure 5: Calculated pressure-volume results for high-spin (Mg,Fe)O

The calculated results for pure and 25% Fe-bearing ferropericlase liquids at 3000, 5000, and 8000 K are shown along with the 3000 K solid results (grey symbols). The concentrations of 6.25, 12.5, and 18.75% Fe at 5000 K are shown by triangles, crosses, and squares, respectively. Note that the pressure values (diamonds) for ferropericlase containing 25% Fe are mostly shifted upwards relative to the pure values (circles and equation of state fit curves).

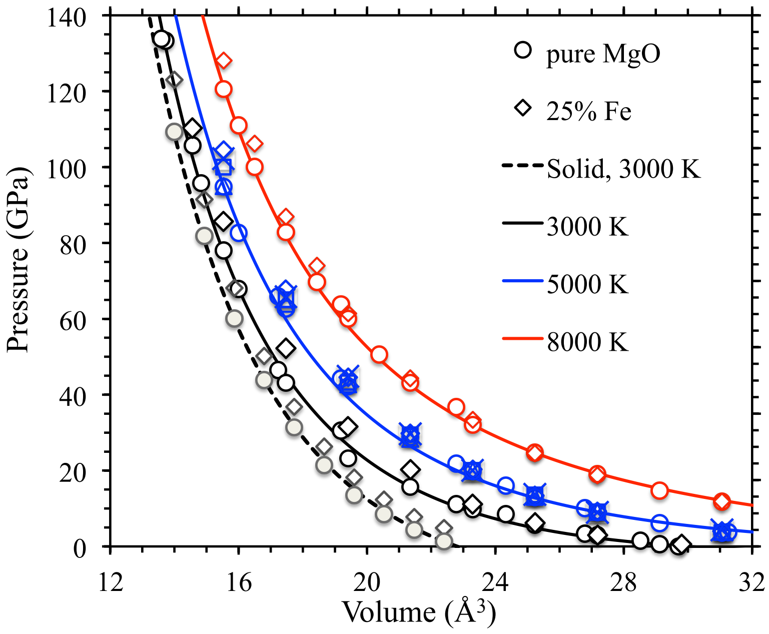

Supplementary figure 6: Additional density comparisons

The liquid and solid densities along the 4000 K isotherm are compared for additional concentrations (3.125, 5.0, and 17.75% Fe) with more crossovers marked.

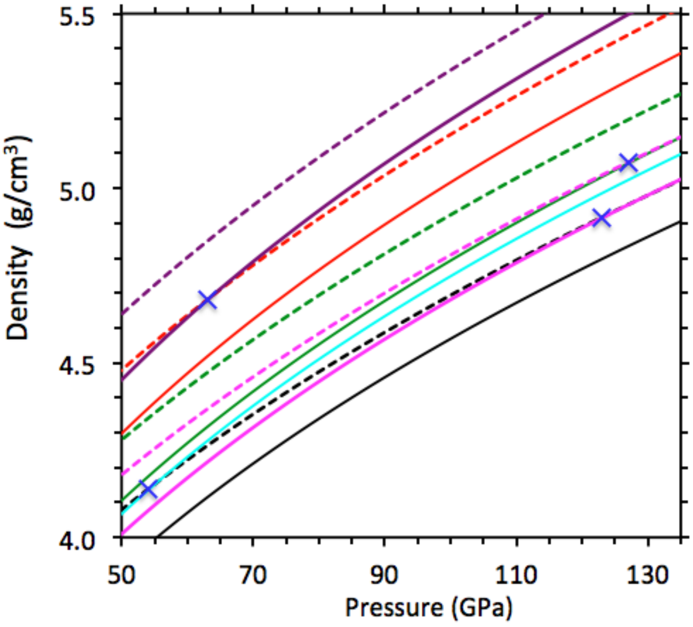

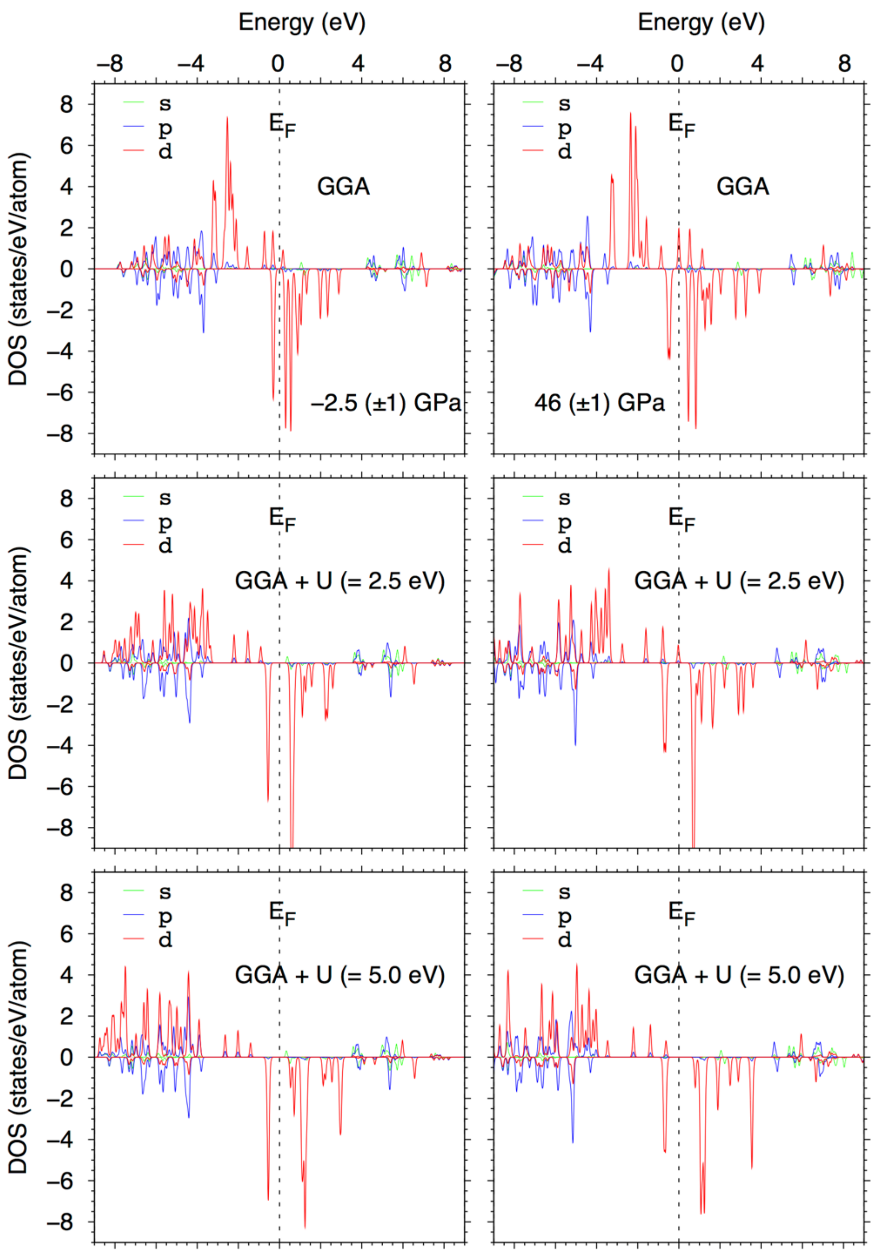

Supplementary figure 7: Electronic density of states (DOS) for ($Mg_{0.8125}$,$Fe_{0.1875}$)O solid obtained from static simulations using GGA and GGA+U

The left panel shows the results at the near ambient pressure and the right panel shows those at high pressure. The GGA-based DOS at low pressure has a small value at the Fermi level. With GGA+U*,* the electronic structure shows new features, such as opening of the band gap, shifting of the occupied *d*-states to deeper energy, and slight narrowing of the *d*-bands. At high pressure, the system may be semi-metallic (U = 2.5 eV) or insulating (U = 5.0 eV), depending on the value of U. Very high U values may lead to unrealistic scenarios, such as the persistence of band gap at high pressures. Moreover, the effects and applicability of U over most of the lower mantle conditions are yet to be explored.

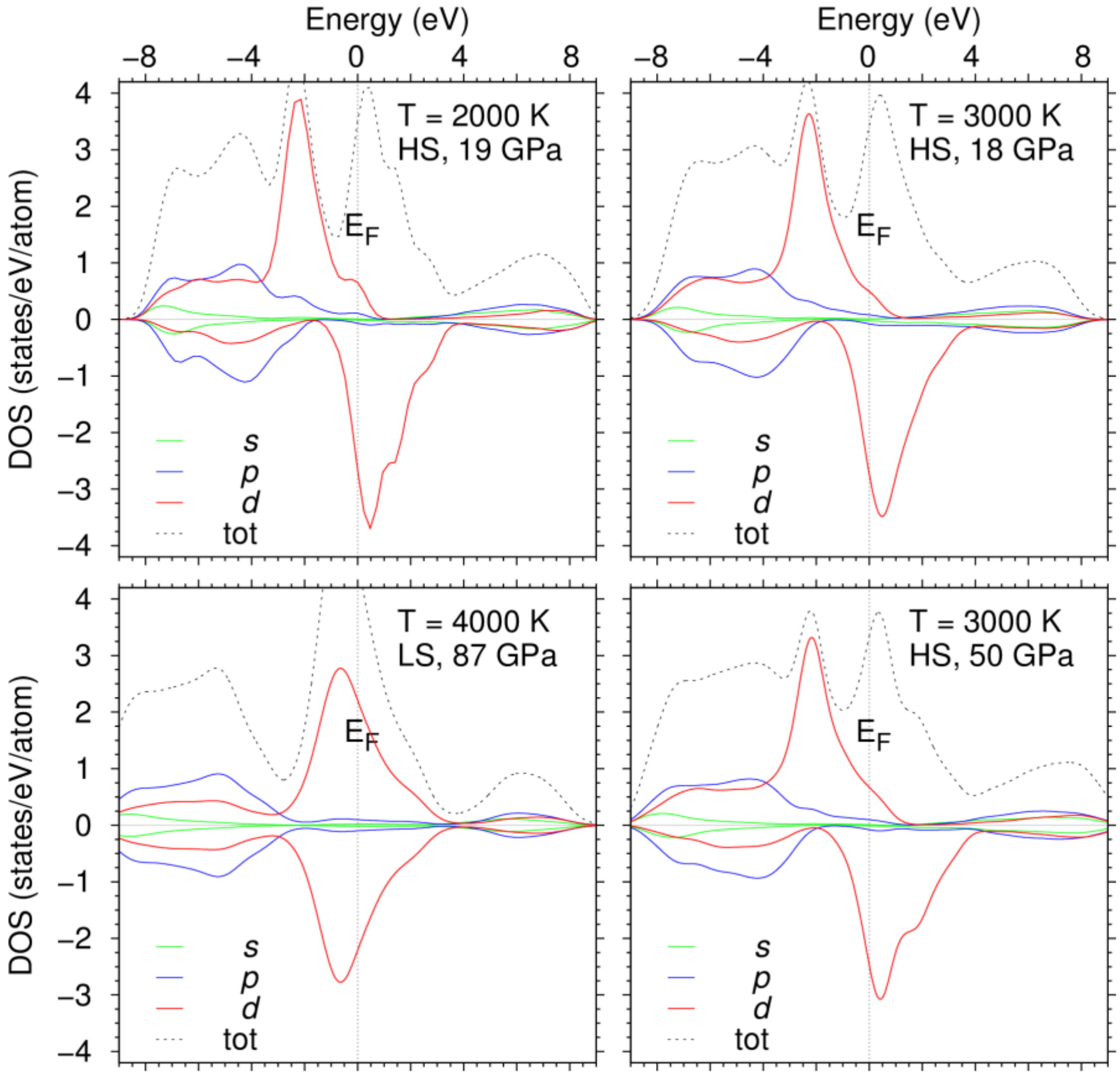

Supplementary figure 8: Representative density of states (DOS) for ($Mg_{0.75}Fe_{0.25}$)O solid at high temperatures

Each DOS result was obtained from the static calculation performed for a snapshot taken from the corresponding first-principles MD simulation (with electronic temperature[7] set at the corresponding MD simulation temperature). HS and LS refer to high- and low-spin states of Fe, respectively. The main electronic contributions around the Fermi level are due to the *d*-electronic states. At a particular volume, temperature causes a small increase in the solid DOS at the Fermi level. At a particular temperature (including 0 K), pressure tends to suppress the solid DOS value at the Fermi level.

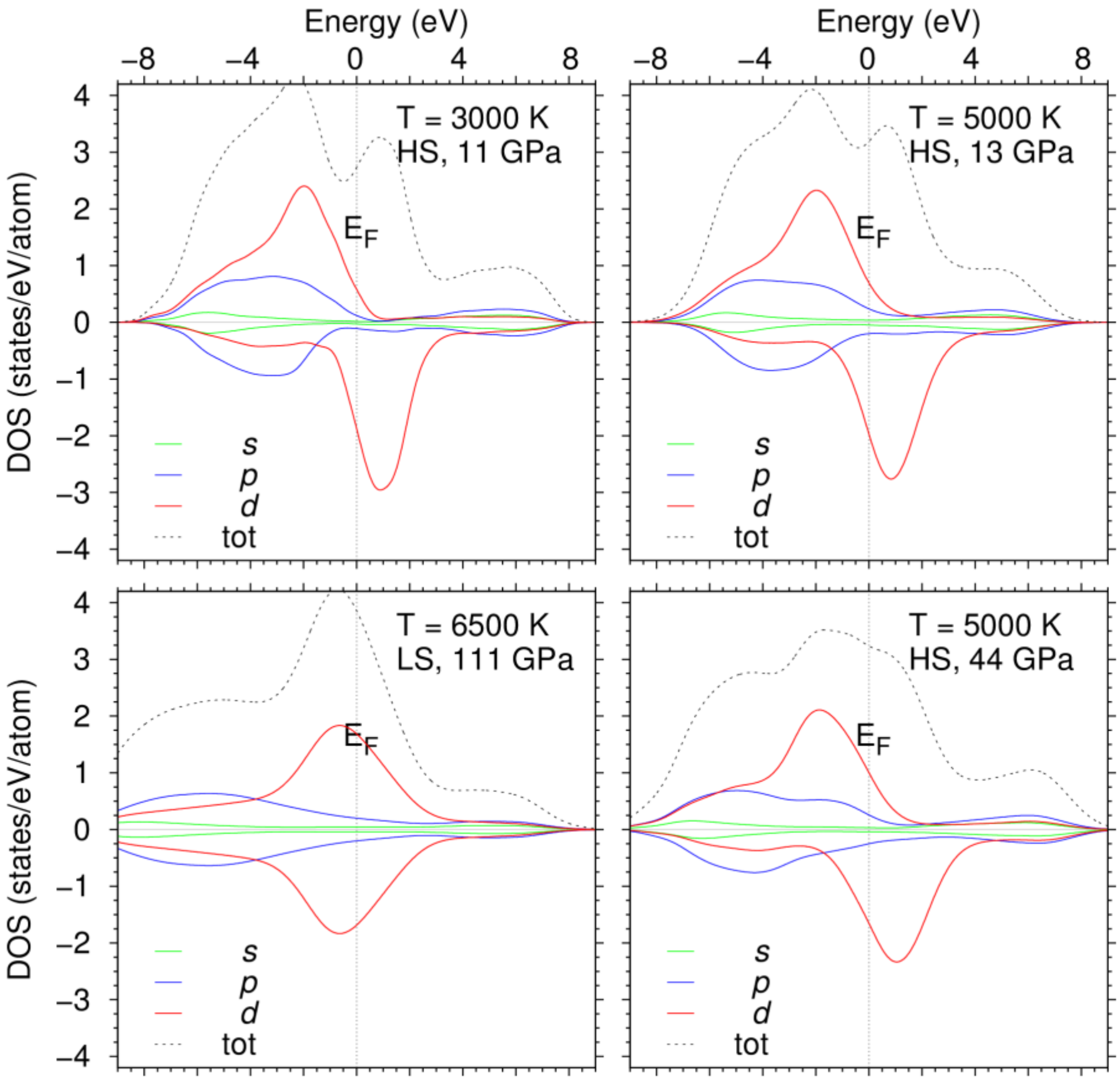

Supplementary figure 9: Representative density of states (DOS) for ($Mg_{0.75}Fe_{0.25}$)O liquid at high temperatures

The changes in the liquid DOS at the Fermi level due to pressure and temperature are small.

Supplementary text: Calculation of Spin Phase Diagrams

The spin phase diagrams for solid and liquid (Mg,Fe)O were calculated by minimizing the Gibb's free energy: $G(P,T,n) = H(P,T,n) - TS$ of all physically possible spin states over wide pressure-temperature range. Here, $H(P,T,n)$ represents the enthalpy computed for each n, defined as $n = n_{LS} / (n_{LS} + n_{HS})$, that is, the fraction of low-spin (LS) states. The entropy term consists of two main contributions[2,8]:

$$\text{magnetic, } S_m = k_B \sum_1^N \left[(1-n) ln(\mu + 1)\right] \quad (1)$$

$$\text{configurational, } S_c = -k_B \sum_1^N \left[n ln n + (1-n) ln(1-n)\right] \quad (2)$$

and also vibrational contributions to some extent[9]. The net contributions to S_c are taken only for the HS and LS iron atoms[8,9].

In the simulations, the value of magnetic moment (μ) for each atom remains 0 for low-spin state and it differs from the ideal value of 4 for high-spin state regardless of GGA or GGA+U. The calculated bulk value of μ is around 3.6 μ_B/Fe atom at the ambient volume and it changes somewhat with compression. Also, a small net moment could originate from strong hybridization of the oxygen p and iron d states, particularly at compressed conditions. At finite pressure-temperature conditions, including magnetic contributions to the entropy from all atoms obtained directly from the simulations makes more sense than considering only Fe atoms.

Typically, the magnetic entropy can be calculated as:

$$S_m = k_B \sum_1^{N_{Fe}} \left[(1-n)\ln((2S+1))\right], \quad (3)$$

where $S = 2$ for HS, $S = 0$ for LS, and N_{Fe} is the number of Fe atoms. In otherwise ideal situation, one should consider the orbital degeneracy (3) of the t_{2g} states to the entropy for HS:

$$S_m = k_B \sum_1^{N_{Fe}} \left[(1-n)\ln\left(3(2S+1)\right)\right] \quad (4)$$

Previous computational studies[2,8,9] have used both the equations 3 and 4 to calculate the magnetic entropy contributions.

References:

1. Mao, Z., Lin, J. -F., Liu, J. & Prakapenka, V. B. Thermal equation of state of lower mantle ferropericlase across the spin crossover. *Geophys. Res Lett.* 38, L23308 (2011).

2. Holmström, E. & Stixrude, L. Spin Crossover in ferropericlase from first-principles molecular dynamics. *Phys. Rev. Lett.* 114, 117202 (2015).

3. Komabayashi, T., Hirose, K., Nagaya, Y., Sugimura, E. & Ohishi, Y. High-temperature compression of ferropericlase and the effect of temperature on iron spin transition. *Earth Planet. Sci. Lett.* 297, 691-699 (2010).

4. Lin J.-F, Vanko G., Jacobsen S. D., Iota V., Struzhkin V. V., Prakapenka V. B., Kuznetsov A., and Yoo C.-S. Spin transition zone in earth's lower mantle. *Science* 317, 1740 (2007).

5. Rollmann G., Rohrbach A., Entel P., and Hafner J. First-principles calculation of the structure and magnetic phases of hematite. *Phys. Rev. B* 69, 165107 (2004).

6. Holmström, E. & Stixrude, L. Spin Crossover in liquid (Mg,Fe)O at extreme conditions. *Phys Rev B.* 93, 195142 (2016).

7. Mermin, N. D. Thermal properties of the inhomogeneous electron gas. *Phys. Rev. A* 137, A1441 (1965).

8. Tsuchiya, T., Wentzcovitch, R. M., da Silva, C. R. S., and de Gironcoli, S. Spin Transition in Magnesiowüstite in Earth's Lower Mantle. *Phys. Rev. Lett.* 96, 198501 (2006).

9. Wu, Z., Justo, J. F., da Silva, C. R. S., de Gironcoli, S. & Wentzcovitch, R. M. Anomalous thermodynamic properties in ferropericlase throughout its spin crossover transition. *Phys. Rev. B* 80, 014409 (2009).
